# Communicability of Cerebrospinal Meningitis

**Published:** 1889-12-07

**Authors:** 


### The Practitioner's Outlook and Retrospect.

[*The Editor will be glad to receive offers of co-operation and contributions from members of the profession. There is no doubt that much valuable material full of interest and instruction is at present lost to science. This is largely so in the case of* (1) *the younger and less-known members of the hospital staff,* (2) *those connected with cottage hospitals, and* (3) *the great body of medical practitioners not attached to any institution. The co-operation of all is cordially invited to enable the Editor to make* The Hospital *of the utmost possible use and value to the profession. Specialists willing to review books on their special subjects are invited to communicate this fact at once. All letters should be addressed to* The Editor, The Lodge, Porchester Square, London, W.]

### MEDICINE.

### COMMUNICABILITY OF CEREBROSPINAL MENINGITIS.

The recent reappearance of this disease in England lends a value to any clear evidence as to its contagious character, which asserted by Aitken and Bristow, is denied by F. Roberts. When attacking almost simultaneously numbers of individuals, as soldiers in barracks, it may be, and often has been, referred to quasi-malarial or other local conditions, to which all have alike been exposed, but the account published in the *Berlin. Klin. Wochensch.*, by Dr. Kohlmann, of a series of cases, in an otherwise healthy population, every one traceable directly to infection from a single originally imported case, is conclusive, besides giving a clue to the incubation period. A girl, æt. seventeen, the daughter of poor parents at Remagen, but at that time in domestic service at Godesburg, was sent home ill on August 25th 1888. The disease proved to be cerebrospinal meningitis, of which she died on September 2nd, being buried on September 6th. From her arrival till her burial she lay in the only room of the cottage. Her father and brother borrowed for the funeral coats, etc., from a neighbouring farmer, F. M., and a lad who, though living in the village, worked at an ornamental tile factory some three miles off. On December 20th, the farmer, æt. sixty-one, was attacked with cerebrospinal meningitis, and his son W. M., æt. eighteen, on January 9th, 1889. Both recovered, but on January 16th his daughter J. M. contracted it in a severe form and died in 48 hours. V., a married woman, æt. twenty-nine, who came in several times each day to attend to her friend J. M., and on one occasion supported her head while Dr. Kohlmann examined his patient, was seized on the eighth day with rigors, headache, etc., which six days later developed into cerebrospinal meningitis, but ultimately she, too, recovered. All these persons were healthy and robust.

The second series of cases included B. B., the factory lad, æt. fourteen, fairly healthy, but not strong, and his mother, æt. fifty-seven, who lived at some distance from the M. family, with whom they had no acquaintance or communication. B. B. was seized on January 29, and died in seventy-two hours, while his mother was attacked on February 2 with pneumonia, complicated with cerebrospinal symptoms (or *vice versâ*), and succumbed to œdema of the lung after seven days. Their cottage was clean and roomy, though damp. In the case of each family the clothes, which were retained for several days after the funeral in the close, infected apartment of the first sufferer, were the only possible cause of the disease, which was and remained otherwise unknown in the district. The case of Mrs. V. suggests an incubative period of about a week, which is confirmed by those of Johanna M. and Mrs. B. The long period that elapsed between the primary case and the first of each secondary series is explained by Dr. K. by the usual conditions of the dwellings of the working classes in winter, when the disease, almost confined to such, is most prevalent—viz., warmth and defective ventilation, with cooking, washing, etc., carried on in the common living room, where they spend more of their time than in summer, and are therefore more influenced by what he calls the "house climate"—an important factor in the development of diphtheria, as well as of the disease under consideration.

